# Assessment of semen parameters in consecutive ejaculates with short abstinence period in oligospermic males

**DOI:** 10.5935/1518-0557.20210073

**Published:** 2022

**Authors:** Vinuta Kulkarni, Pankaj Kaingade, Namrata Kulkarni, Tejal Bhalerao, Amar Nikam

**Affiliations:** 1 Assistant Professor, Department of Obstetrics and Gynecology, SDM Medical college, Dharwad, Karnataka, India; 2 Laboratory Director & Chief Embryologist, Sunanda IVF & Fertility Clinic, Kolhapur, Maharashtra, India; 3 Associate Professor, Department of Obstetrics and Gynecology, SDM Medical college, Dharwad, Karnataka, India; 4 Senior Embryologist, Shri Chaitanya Fertility Clinic, Mumbai, Maharashtra, India; 5 Senior Embryologist, Sunanda IVF & Fertility Clinic, Kolhapur, Maharashtra, India

**Keywords:** semen analysis, short abstinence, consecutive ejaculate, oligospermic males, DNA fragmentation

## Abstract

**Objective:**

Human sperm parameters varies widely among men and even between consecutive samples in the same individual with respect to their concentration, motility, morphology, and DNA fragmentation. Less is known about the characteristics of sperm in short abstinence periods. Hence, the current study was conducted to determine the influence of consecutive ejaculate on above parameters after short abstinence period in oligospermic males.

**Methods:**

This observational study was conducted from January 2018 to February 2019 and included 67 men undergoing primary infertility treatment at the SDM Fertility Centre, Dharwad, India. The first semen sample was provided after an abstinence period of 2-7 days, while the second sample was collected 1-3 h after the first. The two consecutive semen samples were analyzed according to the 2010 WHO criteria for semen analysis and their parameters were compared. Sperm DNA fragmentation was also measured.

**Results:**

Most of the participants were aged of 31 to 40 years (68.6%). The majority of them had the second sample collected after a 1-hour interval (88%); 10.4% of the subjects had the second sample collected after a 2-hour interval; the remaining 1.4% had the second sample collected after a 3-hour interval. Mean concentration (mill/ml), total motility, and progressive motility (%) were significantly higher in the second sample (*p*<0.05). The second sample also showed lower DNA fragmentation than the first ejaculate sample.

**Conclusions:**

Our study inferred that consecutive semen samples collected 1-3 hours apart might have a role in managing subfertility in oligospermic males. Further research, possibly a randomized clinical trial, is needed to explore this association.

## INTRODUCTION

Infertility among couples is increasing, with male factor accounting for 40-50% of all cases (Brugh & Lipshultz, 2004; Hirsh, 2003), affecting about 7% of the male population (Lotti & Maggi, 2015). Male fertility is influenced by factors related to semen quality and quantity. Likewise, abnormal semen quality and sexual dysfunction are contributing factors in about half of subfertile couples (Bayasgalan *et al*., 2004; Sharma, 2017). Semen analysis is a standard diagnostic test performed routinely at IVF laboratories for couples dealing with infertility (Lu *et al*., 2010; Wang & Swerdloff, 2014).

Earlier studies have shown that conventional sperm parameters (concentration, motility, and morphology) vary signiﬁcantly between different individuals and even between consecutive samples taken from the same man (Gosálvez *et al*., 2011; Lewis, 2007). Furthermore, the 2010 WHO manual for sperm analysis recommends that the length of abstinence should range between 2-7 days before diagnostic semen analysis (WHO, 2010). Previously, it was assumed that sperm count was inversely proportional to the duration of intercourse, as sperm concentration, counts, and volume were seen to decline dramatically with sequential ejaculation (Levin *et al*., 1986; Oldereid *et al*., 1984). However, there is a lack of consensus on the exact influence of the abstinence period on sperm parameters. The impact of sexual abstinence on conventional sperm parameters is still debatable (Comar *et al*., 2017; Gosálvez *et al*., 2011; Sánchez-Martín *et al*., 2013). It has been often reported that ejaculates obtained after a short period of abstinence are of poor and unacceptable quality (Barash *et al*., 1995). Consecutive second ejaculate samples collected within one to three hours of the first were also found to yield better semen quality (volume, count, and motility), particularly for parameter total motile sperm count in assisted reproductive technology cycles (Alipour *et al*., 2017; Bahadur *et al*., 2016; Manna *et al*., 2020; Ortiz *et al*., 2016).

Furthermore, a few studies have also shown that short abstinence periods lead to decreased incidence of sperm DNA fragmentation and increased pregnancy rates after assisted reproductive technology treatment (Gosálvez *et al*., 2011; Sánchez-Martín *et al*., 2013).

There is little information available today on the quality of semen ejaculates after very short abstinence intervals, particularly in the Indian population. Only a handful of published studies have looked into the relationship between consecutive ejaculates and semen parameters. Hence, the present study was conducted to determine the influence of consecutive ejaculates on semen parameters after short abstinence periods.

## MATERIAL AND METHODS

The Ethics Committee of our institution approved this study. All participants gave consent in written to having their data published in this study. This observational study conducted from January 2018 to February 2019 included 67 men undergoing primary infertility treatment at the SDM Fertility Centre, Dharwad, Karnataka, India.

The mean age of the oligospermic patients was 33.58±4.78 years. Patients with azoospermia, exposure to toxicants (cigarettes, drugs), or on medication (hormones, vitamins, supplements) were excluded from the study.

The participants were instructed to abstain from sex for 2 to 7 days. All semen samples were produced by masturbation within the clinic environment. This was followed by the collection of consecutive semen samples after a short time period of 1 to 3 hours, dictated by how long it took for the subjects to produce the sample. The study reported semen characteristics (volume, concentration, motility & DNA fragmentation index) for the initial and consecutive ejaculate samples only.

Semen analysis: Semen samples (n=67) were collected in sterile containers by masturbation. Complete liquefaction of the sample was done for 30 to 60 minutes (37^o^C) and the volume was determined using a graduated tube (accurate to 0.2 ml). Semen analysis was performed based on the World Health Organization guidelines 5^th^ Edition 2010; reference limit values were taken from the same publication (WHO, 2010). Concentration was determined using a Makler chamber (Sefi-Medical Instruments, Haifa, Israel).

Sperm DNA fragmentation for semen samples (n=17) was evaluated using the Qwik Check DFI test assay using conventional bright-field microscopy. The methodology behind DNA fragmentation is based on the Sperm Chromatin Dispersion (SCD) test (Fernández *et al*., 2005). Here, the intact unfixed spermatozoa are initially treated with an acid that denatures the double-stranded DNA of the sperm head to single-stranded DNA. Next, the lysing solution removes most of the nuclear proteins and in the absence of massive DNA breakage, produces nucleoids with large halos of disseminated DNA "loops" emerging from the central core. Conversely, the nucleoids of spermatozoa with fragmented DNA either do not show a dispersion halo at all or the halo is minimal. DFI is expressed as the percent of spermatozoa with DNA fragmentation in relation to the total amount of sperm. A minimum of 250-300 spermatozoa for each sample were counted.

### Statistical methods

The differences in sperm parameters between consecutive ejaculates were determined by the t-test for paired samples. Data Analysis was performed using SPSS Software version 22 and a *p*-value of <0.05 was considered to be statistically significant. All data are expressed in mean±SD.

## RESULTS

Most of the 67 participants were aged 31 to 40 years (68.65%) and 8.95% were aged > 40 years. The majority of the participants (88%) had the second sample collected after a 1-hour interval; 10.44% had the second sample collected after a 2-hour interval; and 1.4% had the second sample collected after a 3-hour interval (Table 1).

**Table 1.** Distribution of patients according to age and abstinence interval (n=67).

	Frequency	%
**Age** 21 – 30 31 – 40 40 – 50 Total	15 46 6 **67**	22.3 68.6 8.9 **100**
**Interval (hours)** 1 2 3	59 7 1	88 10.4 1.4

Mean abstinence period (days): 4.0±0.9 (3-7)

**Figure 1:**
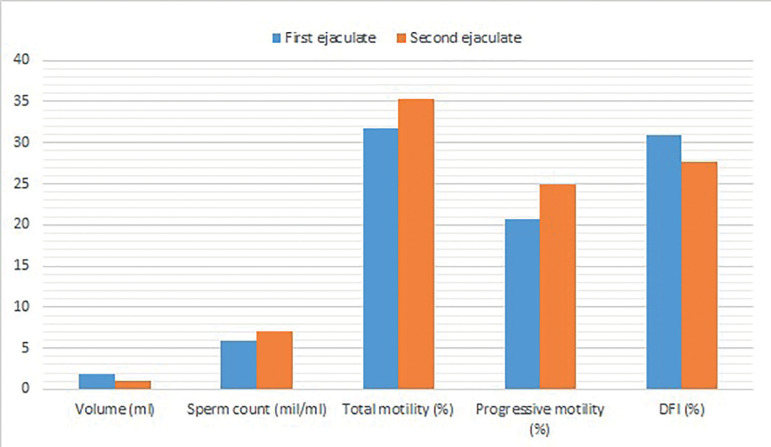
Mean values of sperm parameters (Volume (ml), Sperm count (mil/ml), Total motility (%), Progressive motility (%) and DFI (%) for the initial first (blue bar) and second (orange bar) ejaculates.

The second raw ejaculates demonstrated significant increases in sperm concentration (7.07±3.83 *vs*. 5.92±3.22, *p*<0.05), total motility (35.38±8.60 *vs*. 31.70±8.47, *p*<0.05), and progressive motility (24.98±6.69 *vs*. 20.71±6.95, *p*<0.05) then first ejaculates. Furthermore, the second ejaculates showed lower DNA fragmentation rates than the first ejaculates (27.62±10.10 *vs*. 30.97±11.18, *p*<0.05). Also, as expected, volumes decreased in the second ejaculate samples when compared with the first (1.11±0.56 *vs*. 1.89±0.97, *p*<0.05) (Table 2 and Figure 1).

**Table 2.** Comparison of semen quality parameters between first and second ejaculate with short abstinence period in oligospermic males.

Parameters	First Ejaculate	Second Ejaculate	*p* value
Volume (ml)	1.89±0.97	1.11±0.56	< 0.05
Concentration (mil/ml)	5.92±3.22	7.07±3.83	< 0.05
Total motility (%)	31.70±8.60	35.38±8.47	< 0.05
Progressive motility (%)	20.71±6.95	24.98±6.69	< 0.05
DNA Fragmentation (%)(n=17)	30.97±11.18	27.62±10.10	< 0.05

Values are mean ± standard deviation. *P* value indicates that a significant difference exists between the first and second ejaculate.

## DISCUSSION

Semen examination is an important diagnostic method in determining the male partner's infertility in a couple. To achieve a reliable interpretation of semen characteristics, men are asked to abstain from sex for 2-7 days. The same is recommended in the WHO 2010 criteria used as a reference for semen analysis in this study (WHO, 2010). Many studies have shown that semen volume, sperm concentration, and total sperm count increase as the duration of abstinence is extended up to 4-7 days (Alipour *et al*., 2017; Agarwal *et al*., 2016; Raziel *et al*., 2001). Hence, it was decided that the same protocol would be adopted in our study.

A few researchers have suggested that a second consecutive sperm ejaculate after a short abstinence period (30 minutes-3 hours) might improve sperm quality in subfertile men (Alipour *et al*., 2017; Bahadur *et al*., 2016; Juárez-Bengoa *et al*., 2010; Sugiyam *et al*., 2008; Zhai *et al*., 2011). Similarly, we decided to compare sperm quality between the first ejaculate and the second consecutive ejaculate obtained after a 1-3-hour interval.

Our study found a significant improvement in sperm concentration, total motility, and progressive motility in the second sample, even though there was a slight change in volume. Also, sperm DNA fragmentation was significantly lower in the second sample (Table 2 and Figure 1). Many other studies have compared sperm parameters in consecutive ejaculates with variable outcomes. Alipour *et al*. (2017) found lower semen volume, sperm concentration, total sperm counts, and total motile counts. However, higher percentages of motile spermatozoa with higher velocity and progressiveness were detected in samples obtained after 2h. In a study by Barash *et al*. (1995), statistically significant improvement was shown in sperm cell motility and in motile count after swim-up. But no improvement was demonstrated in sperm density or morphology. Volume also decreased significantly from the first to the second ejaculate. Bahadur *et al*. (2016) observed a drop in semen volume, but also a significant improvement in sperm motility and normal morphology. Many other authors had findings similar to the ones of our study (Gosálvez *et al*., 2011; Goss *et al*., 2019; Ortiz *et al*., 2016; Sugiyam *et al*., 2008). However, in the study by Mayorga-Torres *et al*. (2016), in which four consecutive semen samples were collected every two hours, significant decreases in conventional semen parameters were observed at each evaluation (*p*<0.05). Here, abstinence was found to have no influence on sperm parameters. Furthermore, Scarselli *et al*. (2019) determined that semen volume was lower in the second sperm retrieval, while sperm concentration, motility, and morphology were similar in the two groups.

Furthermore, our findings on sperm DNA fragmentation were in agreement with recent studies. Hussein *et al*. (2008) detected a significant improvement in DNA integrity in the second semen sample collected after 1-3 h compared with the first sample of 20 infertile patients with idiopathic OAT. Shen *et al*. (2019) also reported that the sperm DNA fragmentation index was slightly lower (*p*<0.05) in semen samples collected after 1-3 h of abstinence compared with the index seen in spermatozoa collected after a longer period of abstinence (3-7 days).

All abovementioned studies inferred that short abstinence was associated with decreased ejaculate volume. This may be attributed to insufficiency of the accessory sex glands to make an adequate contribution to the ejaculate volume, particularly the seminal vesicles and the prostate gland, known as primary contributors (Ayad *et al*., 2018). Another consistent observation was the increase in total motility of sperm. However, the exact mechanism by which ejaculatory abstinence affects semen motility is unknown. It has been suggested that reduction in the storage period within the epididymis may minimize the exposure of unejaculated spermatozoa to motility inhibitory factors and enzymes released from the degenerating cells within the same microenvironment (Valsa *et al*., 2013). Furthermore, extending the abstinence time may also enhance susceptibility of unejaculated spermatozoa to recurrent genital heat exposure, causing detrimental changes. Therefore, reducing the abstinence period may minimize the frequency and time span of heat exposure, thereby leading to improved motility (Ayad *et al*., 2018; Wechalekar *et al*., 2010).

The key limitations of our study include its observational design and the convenience sampling method that enables the introduction of selection and ascertainment bias. Also, ours was a single-center study that enrolled only individuals seeking care at the fertility clinic, which limits the generalizability of study findings. Our sample size also limited the external validity of the study. However, collecting replicate semen samples from the same individuals is probably an effective approach to controlling for confounding factors. Other potential limitations of our study include: (1) other factors besides ejaculatory abstinence may account for the effects observed; and (2) our inability to determine the confounding factors and other lifestyle-related factors such as age, physical activity levels, dietary habits, nutritional intake, and general lifestyle habits of the included population might lead to confounding bias. The female infertility factor was also ruled out from this study. In spite of these limitations, the study significantly contributed to the existing literature on consecutive ejaculation. Future research must include multicenter randomized trials so that the factors connected with abstinence, semen quality, and consecutive ejaculates are thoroughly understood.
